# *Giardia* spp. and the Gut Microbiota: Dangerous Liaisons

**DOI:** 10.3389/fmicb.2020.618106

**Published:** 2021-01-12

**Authors:** Elena Fekete, Thibault Allain, Affan Siddiq, Olivia Sosnowski, Andre G. Buret

**Affiliations:** ^1^Department of Biological Sciences, University of Calgary, Calgary, AB, Canada; ^2^Inflammation Research Network, University of Calgary, Calgary, AB, Canada; ^3^Host-Parasite Interactions, University of Calgary, Calgary, AB, Canada

**Keywords:** Giardia duodenalis, microbiota, biofilm, polymicrobial infections, probiotics, giardiasis, tissue barrier, pathobiont

## Abstract

Alteration of the intestinal microbiome by enteropathogens is commonly associated with gastrointestinal diseases and disorders and has far-reaching consequences for overall health. Significant advances have been made in understanding the role of microbial dysbiosis during intestinal infections, including infection with the protozoan parasite *Giardia duodenalis*, one of the most prevalent gut protozoa. Altered species composition and diversity, functional changes in the commensal microbiota, and changes to intestinal bacterial biofilm structure have all been demonstrated during the course of *Giardia* infection and have been implicated in *Giardia* pathogenesis. Conversely, the gut microbiota has been found to regulate parasite colonization and establishment and plays a critical role in immune modulation during mono and polymicrobial infections. These disruptions to the commensal microbiome may contribute to a number of acute, chronic, and post-infectious clinical manifestations of giardiasis and may account for variations in disease presentation within and between infected populations. This review discusses recent advances in characterizing *Giardia*-induced bacterial dysbiosis in the gut and the roles of dysbiosis in *Giardia* pathogenesis.

## Introduction

The Protozoan *Giardia duodenalis* is one of the most common human parasitic enteropathogens worldwide, infecting on average 2% of adults and 8% of children in developed countries and up to 33% of individuals in the developing world (Dunn and Juergens, 2020). *Giardia* infections are also common in livestock and companion animals and are responsible for significant global health and economic burdens. Infective *Giardia* cysts are typically ingested *via* contaminated food or water or *via* the fecal-oral route. Excystation begins in the stomach, where cyst walls are weakened by host proteases and acidic pH, and is completed in the upper small intestine with the combined action of host and *Giardia* proteases. Vegetative trophozoites are released into the small intestinal lumen, where they divide by binary fission and attach to the host epithelium *via* a ventral adhesive disk. The life cycle is completed when trophozoites detach and begin to move further along the GI tract, where they are exposed to bile, and initiate encystation to form new cysts that are excreted into the environment. Acute giardiasis typically causes diarrhea, abdominal pain, nausea, intestinal malabsorption, and weight loss. However, symptoms can occur along a broad spectrum, and asymptomatic infection is common (Halliez and Buret, 2013). *Giardia* may even have protective effects against diarrheal disease in some settings, particularly in children exhibiting polymicrobial infections. In contrast, *Giardia* infection has also been associated with development of post-infectious complications including irritable bowel syndrome (IBS) and chronic fatigue and has been associated with cognitive defects and stunted growth in children (Halliez and Buret, 2013; Allain and Buret, 2020). The precise mechanisms that govern *Giardia* pathogenesis are incompletely understood, however, recent research has emphasized important roles for the microbiota.

The intestinal microbiota plays a critical role in homeostasis of the gut and overall health and is frequently found to be altered during gastrointestinal disease. *Giardia* interacts both directly and indirectly with the microbiome, and through these interactions can modulate host metabolism, immune responses, pain signaling, and the mucus barrier, all of which may have systemic effects that potentially persist even after parasite clearance. Functional and compositional changes to the intestinal microbiota have been demonstrated during the course of *Giardia* infections, including disruption of the microbial biofilm structure, altered virulence in commensal species, and altered species abundance and diversity. In turn, the disrupted microbiota plays a role in *Giardia* pathogenesis, influencing colonization resistance, immune responses, and brush border defects. Future research is required to define the role of the microbiota in other aspects of *Giardia* pathogenesis.

Understanding the role of the intestinal microbiota in *Giardia* pathogenesis will help to develop therapeutics for a broad range of disorders that implicate disruptions of gut commensal microbes. Recent research has focused on the use of probiotics in the prevention and treatment of giardiasis, as several probiotic microbes have been found to have anti-*Giardia* effects *in vitro* and *in vivo*. Dietary interventions may also contribute to therapy, as the diet has been shown both to alter the microbiome and to influence *Giardia* pathogenesis. Identification of *Giardia*-associated microbiome signatures has also been attempted, which may allow for more targeted studies of the roles of specific commensals during *Giardia* infection. In this review, we provide state-of-the-art information on the interactions between *Giardia* and the gut microbiota, and their roles in the pathogenesis of giardiasis.

## The Intestinal Microbiome

### Structure and Composition of the Intestinal Microbiota

The intestinal microbiome, encompassing bacteria, viruses, protozoa, and fungi, is a complex and dynamic community of microorganisms that exists in close association with the host intestinal mucosa. Interactions between the gut microbiome and the host have been shown to be important for health and homeostasis. In particular, the bacterial component of the microbiome may play critical roles in the development and persistence of intestinal and extra-intestinal disorders and will be the focus of this review (Carding et al., 2015; Shreiner et al., 2015; Nagao-Kitamoto et al., 2016; Buret et al., 2019).

Dysbiosis, broadly defined as a maladaptive alteration to the bacterial species composition, diversity, or abundance relative to bacterial communities in a healthy individual, has been demonstrated in multiple diseases and disorders of the gastrointestinal tract, including infectious diseases (i.e., bacterial, viral, fungal, and parasitic), irritable bowel syndrome (IBS), inflammatory bowel disease (IBD), and intestinal cancers (Carding et al., 2015; Shreiner et al., 2015; Iacob and Iacob, 2019). The intestinal microbiome plays a major role in the development and maintenance of gut homeostasis, and therefore its disruption may contribute both to initiation and progression of intestinal disease (Wu and Wu, 2012; Lin and Zhang, 2017). Important functions of the microbiome include digestion and absorption of nutrients, immune maturation and modulation, regulation of host cell proliferation and function, modulation of intestinal permeability, transit, and neurotransmission, and defense against opportunistic pathogens *via* niche exclusion (Lynch and Pedersen, 2016). High microbial diversity is a hallmark of the microbiota’s functional redundancy and contributes to its stability, resilience, and adaptability (Tuddenham and Sears, 2015).

Although the composition and abundance of bacterial taxa are highly variable between individuals, it has been suggested that healthy individuals share a “core” microbiota. Recently, the MiBioGen consortium initiative launched a meta-analysis project to better characterize the human microbiome (Wang et al., 2018). In humans, the most prevalent phyla include Firmicutes and Bacteroides, followed by Actinobacteria, Proteobacteria, and Verrucomicrobia (Lozupone et al., 2012; Tuddenham and Sears, 2015). Within these phyla, the specific species composition and their relative abundances can vary dramatically, influenced by genetics, diet, immune status, sex, and age. Microbiota composition also varies throughout an individual’s intestinal tract, with dramatic differences between the intestinal mucosa and luminal environment, and longitudinally along different segments of the GI tract (Lozupone et al., 2012; Hollister et al., 2014; Tuddenham and Sears, 2015). Although the adult intestinal microbiota is generally stable, significant shifts may occur due to antibiotic treatment, intestinal infection, or with long-term changes to the diet (Tuddenham and Sears, 2015).

In the small intestine, environmental conditions are generally harsh due to rapid transit times and the periodic influx of digestive enzymes, bile, and stomach contents. As a result, bacterial populations tend to be more dynamic, but less diverse, and with lower overall biomass than in the large intestine (Kastl et al., 2020). Bacterial population density varies along the small intestine, increasing from approximately 10^4–5^ CFU/ml in the duodenum to 10^7–8^ CFU/ml in the distal ileum (Kastl et al., 2020). Community composition also varies due to changing physical conditions, such as oxygen and pH gradients, with the proportion of Gram positive to Gram negative bacteria and the abundance of facultative anaerobic and strict anaerobic species increasing from proximal small intestine to the distal small intestine and colon (Tropini et al., 2017; Kastl et al., 2020). Genera commonly found in the small intestine include *Lactobacillus*, *Clostridium*, *Staphylococcus*, *Streptococcus*, and *Bacteroides* (Hayashi et al., 2005; Wang et al., 2005; Ahmed et al., 2007). In the colon, the luminal environment tends to be more stable, and as a result, diversity and overall abundance of microbes is higher than in the small intestine, reaching up to 10^11–12^ cells/g of luminal contents (Guarner and Malagelada, 2003). Mucinophilic bacteria, such as *Akkermansia muciniphila* and certain *Bacteroides* species, have been identified in the outer mucus layers adjacent to the lumen, while closer to the mucosa, oxygen gradients select for more aerotolerant taxa such as Proteobacteria and Actinobacteria (Tropini et al., 2017; Flynn et al., 2018). The proximal colonic mucosa tends to harbor facultative anaerobes including *Actinomyces* and *Enterobacteraceae*, and aerobic *Pseudomonas*. The distal mucosa is more likely to host strict anaerobes including *Porphyromonas*, *Anaerococcus*, *Finegoldia*, and *Peptoniphilus* (Flynn et al., 2018).

Commensal intestinal microbes form poly-microbial structures known as biofilms, which facilitate adhesion to the mucus gel and help microbes withstand shear forces. Biofilms create complex and unique microenvironments within a self-produced polymeric extracellular matrix and allow for cooperative interactions within and between microbial species. Sessile biofilm bacteria have radically different physiology than planktonic bacteria, creating distinct differences between the bacterial populations found in the intestinal lumen and those associated with the mucosal surface (de Vos, 2015). Biofilms also tend to have increased tolerance against shifting environmental conditions, antibiotics, and the host immune system compared to free-swimming microbes, making them an important structure for maintaining stable commensal populations (Jefferson, 2004; Burmolle et al., 2014; de Vos, 2015). Disruption of biofilm structure and subsequent release of biofilm bacteria may therefore contribute to intestinal disease development and progression by destabilizing commensal populations, and/or by driving functional and phenotypic changes in commensal microbes (Beatty et al., 2017; Buret et al., 2019).

### Gut Microbiota Dysbiosis in Protozoan Infections

Protozoan infections in humans and other mammals have frequently been associated with dysbiosis (Burgess et al., 2017). Common protozoan infections in humans include *Giardia duodenalis*, *Entamoeba histolytica*, *Cyclospora cayetanensis*, *Cryptosporidium* spp., and *Blastocystis* spp. (Haque, 2007; Hamad et al., 2016; Iebba et al., 2016). Briefly, protozoan parasites, including *Giardia* and *Cryptosporidium*, identified in fecal samples from Columbian children, have been associated with altered microbiome profiles compared to uninfected children (Toro-Londono et al., 2019). Individuals infected with *Entamoeba histolytica*, the causative agent of amebiasis, were found to have a decreased abundance of protective bacteria generally associated with gut health (Verma et al., 2012). *Cryptosporidium* was found to alter microbiota composition in mouse models as well as in primates (Coquerel’s sifakas – *Propithecus coquerel*), and dysbiosis was associated with enhanced parasite growth (Ras et al., 2015; McKenney et al., 2017; Charania et al., 2020). Individuals infected with the protist *Blastocystis* show increased diversity of the gut microbiota in their fecal contents (Audebert et al., 2016; Nieves-Ramirez et al., 2018). Interestingly, increased microbial diversity is generally associated with a healthier gut (Scanlan et al., 2014; Audebert et al., 2016). Overall, there appears to be a close association between protozoan infection and dysbiosis, with unique dysbiotic profiles associated with individual protozoa. More research is needed to determine whether and how dysbiosis may promote parasitic colonization and contribute to pathogenesis; however, dysbiosis is not exclusively linked to increased disease severity. Rather, some cases of protozoan-induced dysbiosis may lead to positive or neutral effects on gut health.

## Role Of The Microbiome In *Giardia* Pathogenesis

### The Gut Microbiota Influences *Giardia* Colonization and Persistence

The intestinal microbiota plays a key role in determining susceptibility or resistance to colonization by *Giardia*. Mice from different commercial breeders, possessing distinct microbiomes, were found to have different susceptibilities to *Giardia* infection (Singer and Nash, 2000). Resistance was found to be transferable to normally susceptible mice *via* co-housing and was eliminated by antibiotic treatment (Singer and Nash, 2000). Further evidence for this role comes from experimental infection of germ-free mice, which show higher fecal cyst counts than conventionally raised mice likely due to both the immaturity of the immune system and the absence of niche competition by commensal microbes (Torres et al., 1992). It is worth mentioning that antibiotic treatment is often required for robust infection of mice with human *Giardia* isolates, suggesting that the microbiome may play a role in establishment and persistence of infection. However, it is important to consider that antibiotic treatment itself may induce dysbiosis. Challenges arise in the study of immune responses, mucosal disruption, bacterial translocation, and pain responses when antibiotics are used, as antibiotic-induced dysbiosis can itself cause disruptions in these areas. Despite these challenges, antibiotics remain a useful tool to study many human isolates of *Giardia in vivo*.

Susceptibility to *Giardia* infection varies significantly among different age groups in humans and in animal models, which is likely due at least in part to age-related shifts in microbiota composition, as well as changes in host immune factors. Several studies have shown the efficacy of using a suckling mouse model to study infection with *Giardia* isolates that do not typically infect adult mice (Hill et al., 1983; Allain et al., 2017, 2018a; Riba et al., 2020). Shifts in the microbiota that occur upon weaning may be responsible for this increased resistance to *Giardia* infection (Hill et al., 1983). Similarly, in human populations, children aged 6 months to 5 years show higher susceptibility to *Giardia* infection than other age groups (Lengerich et al., 1994; Harvey et al., 2020). The microbiota of human infants is distinctly different from that of adults and is continuously evolving through the first several years of life (Lynch and Pedersen, 2016). In the first 6 months of life, maternal milk confers passive immunity. In addition, both *via* direct microbe-trophozoite interactions and by influencing cell signaling pathways and development of the immune system, the microbiota appears to be a critical determinant of susceptibility or resistance to *Giardia* infection.

### Microbiota-Immune System Interactions During *Giardia* Infection

*Giardia duodenalis* is a non-invasive enteropathogen that does not trigger an overt inflammatory response. However, both innate and adaptive immunity are required for control and clearance of *Giardia*. Critical roles have been identified for the cytokines IL-6 and IL-17, as well as for secretory IgA (Langford et al., 2002; Zhou et al., 2003; Dreesen et al., 2014; Dann et al., 2015). CD4+ and CD8+ T cells play important roles in parasite clearance and pathogenesis, respectively (Scott et al., 2004; Keselman et al., 2016). In addition, mucosal mast cells and macrophages appear to play a role in parasite clearance, while nitric oxide and antimicrobial peptides produced by intestinal cells may have direct or indirect cytostatic effects against *Giardia* trophozoites (Belosevic and Daniels, 1992; Aley et al., 1994; Li et al., 2004; Stadelmann et al., 2013; Fink et al., 2019). However, recent findings have demonstrated that *Giardia* is capable of modulating host immunity. *Giardia* may directly cleave host-produced chemokines and cytokines, resulting in dampened immune responses (Cotton et al., 2014a,b; Liu et al., 2018, 2019; Ortega-Pierres et al., 2018; Allain et al., 2019). Furthermore, *Giardia* may avoid host adaptive immunity through the expression of variant-specific surface proteins (VSPs; Langford et al., 2002; Hjollo et al., 2018).

Immune modulation by the microbiota may play a key role in several aspects of *Giardia* pathogenesis. *Giardia* infection is associated with diffuse shortening of the brush border microvilli and disaccharidase deficiencies, which leads to malabsorption and maldigestion of nutrients (Buret et al., 1992; Scott et al., 2004). These effects are driven by CD8+ T cells and transfer of CD8+ T cells from infected mice to naïve animals is able on its own to drive brush border injury and malfunction (Scott et al., 2004; Keselman et al., 2016). Antibiotic treatment was found to attenuate these *Giardia*-induced histopathological alterations and disaccharidase deficiencies by preventing the activation of CD8+ T cells. Interestingly, antibiotic treatment did not affect CD4+ T cell activation. CD4+ T cells play an important role in parasite clearance, and so the reductions in intestinal pathologies in these mice were not due to reduced trophozoite burden (Scott et al., 2004; Keselman et al., 2016). Similarly, histopathological alterations were found to be less severe in germ-free mice than conventional mice infected with *Giardia*. This may be similarly attributed to defects in the host immune response, as germ-free mice are considered to have “immature” immune systems. In comparison to germ-free mice, *Giardia*-infected conventional mice showed higher levels of IgA in the intestinal fluid and higher levels of IgG and IgM in the serum, further corroborating that the microbiota plays important roles in the adaptive immune response during *Giardia* infection and is a driving factor in some aspects of *Giardia* pathogenesis (Torres et al., 2000).

The microbiota can also influence the expression of cytokines during *Giardia* infection. In germ-free mice reconstituted with human colonic microbial communities, mice that received microbes previously exposed to *G. duodenalis* trophozoites (isolate NF) showed increased production of proinflammatory cytokines, as well as proliferation and enlargement of lymphocyte aggregations within the follicles, compared to mice receiving control bacteria. Mice receiving *Giardia*-modified microbial communities showed increased production of IL-6, IL-1*β*, TNF-*α*, and IFN-ɣ, suggesting *Giardia* may alter microbial communities in such a way as to promote a pro-inflammatory response that is driven by the microbes themselves (Beatty et al., 2017).

While the microbiome may modulate immune responses to *Giardia*, *Giardia* in turn may initiate and alter immune responses in ways that contribute to dysbiosis, either through direct effects on microbes or by altering the intestinal microenvironment. *Giardia* has been found to induce Th17 type immune responses, with elevated levels of IL-17 being observed both in mouse models of infection and in Brazilian children infected with *Giardia* (Dreesen et al., 2014; Dann et al., 2015; Cascais-Figueiredo et al., 2019; Singer et al., 2019). IL-17A is an important mediator of anti-microbial responses and plays a role in the transport of IgA across the epithelium and into the intestinal lumen, where it creates an anti-microbial chemical barrier to protect the underlying epithelium (Dann et al., 2015; Singer et al., 2019). IgA also plays a role in *Giardia* clearance, targeting *Giardia* VSPs (Langford et al., 2002; Hjollo et al., 2018). Increased levels of IgA were similarly seen in the saliva and serum of *Giardia*-infected Egyptian children and in mice treated with *Giardia* excretory/secretory products (ESPs; El-Gebaly et al., 2012; Jimenez et al., 2014; Hjollo et al., 2018). In IL-6 deficient mice, Th17 cell development is impaired, whereas IgA production is unaffected, and the clearance of *Giardia* infection is delayed (Bienz et al., 2003; Dann et al., 2015). Increased eosinophil numbers have been observed during *Giardia* infection, and in eosinophil deficient mice, IL-17 levels, as well as serum and luminal IgA levels, are decreased suggesting elevated levels of IL-17 and IgA during *Giardia* infection are dependent in part on eosinophil activity (Jung et al., 2015).

Specific members of the commensal microbiota have been implicated in driving immune responses during *Giardia* infection. Segmented filamentous bacteria (SFB) have been shown to drive IL-17 production (Ivanov et al., 2009; Barash et al., 2017). Mouse colonies that were previously shown to be resistant to *Giardia* infection (Singer and Nash, 2000) were found to have SFB (quantitative PCR on 16S rRNA), while susceptible mice did not, suggesting SFB may play a role in the protective effects of the microbiota against *Giardia* colonization. Following oral inoculation of SFB in germ-free mice, isolated T cells and APC cells from the lamina propria were found to have increased IL-17 expression, while fecal IgA was elevated (Atarashi et al., 2015). These observations are in support of the hypothesis that SFB may contribute at least in part to the characteristic adaptive immune response observed during *Giardia* infection. Interestingly, neonatal mice infected with *Giardia muris* fail to develop a protective IL-17A response and show delayed parasite clearance compared to adult mice (Paerewijck et al., 2019). IL-17A responses and IgA production were only evident after weaning (Paerewijck et al., 2019). Colonization of the GI tract by SFB occurs in rat and mouse hosts at the onset of the weaning process, and thus SFB populations are low in neonates, which may contribute to their inability to mount IL-17 responses and their poor resistance to *Giardia* infection (Koopman et al., 1987; Ericsson et al., 2014). Similarly, Clostridia have been implicated in promotion of regulatory T cell responses (Garland et al., 1982; Keselman et al., 2016; Fink and Singer, 2017). Significant reductions in Clostridia have been observed in *Giardia*-infected mice, which may explain the lack of a significant Treg response typically observed during *Giardia* infection (Barash et al., 2017). However, this appears to be dependent on mouse strain and genetic background. BALB/c mice infected with *Giardia muris* showed a more significant Treg response than C57BL/6 mice and shed more *Giardia* cysts. Interestingly, BALB/c mice showed larger populations of Clostridia than the C57BL/6 mice, further suggesting that presence of certain commensal species may drive specific immune responses (Yordanova et al., 2019). However, in contrast to previous studies, although BALB/c mice showed increased SFB populations, they showed reduced Th17 responses (Yordanova et al., 2019). Complex interactions involving multiple species likely drive microbiome-immune system interactions.

To subvert host immune responses, *Giardia* possesses several mechanisms of immunomodulation. Nitric oxide (NO) is an important antimicrobial compound produced by epithelial cells in the gut. It has broad, non-specific effects against luminal microbes, including *Giardia*. NO is synthesized from L-arginine, which is a primary energy source for *Giardia* trophozoites. Due to *Giardia*’s consumption of L-arginine, NO levels are depleted in the gut during infection, resulting in impaired antimicrobial activity at the mucosal surface (Stadelmann et al., 2012; Allain et al., 2017; Fink and Singer, 2017; Singer et al., 2019). Similarly, *Giardia* can impair host immune response by, at least in part, cleaving cytokines and chemokines such as CXCL1, CXCL2, CXCL3, IL-8, CCL2, and CCL20 in a cysteine protease dependent manner (Cotton et al., 2014a,b; Liu et al., 2018, 2019; Ortega-Pierres et al., 2018; Allain et al., 2019).

The immune system is highly dependent on the microbiota for development and maturation. Studies of immune responses in germ-free mice have revealed that the absence of microbes impacts both innate and adaptive immunity, and thus germ-free mice are considered to have “immature” immune systems. For such studies, the use of antibiotic treatment is a common alternative to germ-free animals, as it allows for the study of animals with a depleted gut microbiota, but fully developed immune system. However, as stated earlier, antibiotic treatment will itself alter immune function by causing dysbiosis, and the types and combinations of antibiotics used must be carefully considered, as each may selectively deplete certain microbial populations, as well as modify the host immune response. Broadly, both antibiotic treated and germ-free animals typically show reduced immune cell populations, altered cytokine production, and disrupted cell signaling pathways. It is important to consider these alterations when studying *Giardia* using antibiotic treated or germ-free models.

### Intestinal Mucus Disruption During Giardiasis: A Role for Microbiota?

The microbiome and the intestinal mucus layers exist in close association. Mucus acts as a physical and chemical barrier that can prevent inflammatory responses to microbes and other potentially harmful luminal contents (Cornick et al., 2015). It also creates a niche environment for commensal species, providing binding sites and acting as a nutrient source when degraded by mucolytic bacteria such as *Bacteroides thetaiotaomicron*, *Akkermansia* sp., or *Ruminococcus* sp., and therefore facilitates microbial colonization of the gut (Schroeder, 2019). Changes to the intestinal mucus gel have frequently been associated with dysbiosis, potentially as a result of altered nutrient or binding site availability, or due to disrupted barrier function, facilitating the translocation of commensal bacteria through the intestinal epithelium and leading to mucosal immune activation (Schroeder, 2019). In turn, dysbiosis may lead to significant changes to the mucus barrier, as the commensal microbiome plays key roles in regulating production and secretion of mucus by intestinal goblet cells and in goblet cell differentiation (Cornick et al., 2015). Indeed, mucus in the large and small intestines of germ-free mice has altered penetrability and composition compared to conventional mice, which can be normalized by microbiota transfer (Johansson et al., 2015). Further, in mouse colonies that vary in microbiota composition, differences in the penetrability of the inner colonic mucus layer to bacteria-sized beads were observed, suggesting variations in commensal populations, and potentially dysbiosis, may predispose individuals to having a more penetrable mucus barrier and therefore increase the risk of pathogen translocation (Jakobsson et al., 2015). Mice with a more penetrable colonic mucus layer showed increased levels of Proteobacteria compared to those with impenetrable mucus (Jakobsson et al., 2015). Interestingly, *Giardia* has been shown to drive expansion of Proteobacteria in mouse models of infection (Barash et al., 2017). In addition, various microbes and microbial products have been found to either inhibit or stimulate mucus production and secretion by goblet cells or to change the chemical composition of mucins, thus altering the intestinal microenvironment and potentially barrier function (Cornick et al., 2015; Schroeder, 2019).

*Giardia* is able to disrupt intestinal mucus (Amat et al., 2017). In mice infected with *G. duodenalis*, significant thinning of the colonic mucus layer can be observed. *In vitro G. duodenalis* can degrade purified human MUC2 mucin in a cysteine protease dependent manner. Together, these findings indicate that, during infection, *Giardia* may cause localized depolymerization of the mucin gel matrix, potentially facilitating the translocation of both the parasite and commensal bacteria. This is consistent with the findings that giardiasis promotes the invasion of commensal bacteria *in vivo* (Halliez et al., 2016; Allain et al., 2017; Beatty et al., 2017; Manko et al., 2017). In addition, *G. duodenalis* strain GS/M alters the expression of both Muc2 and Muc5ac, the predominant secretory mucins expressed in the intestines. Specifically, *in vivo*, Muc2 and Muc5ac expressions were increased in the colon; while in the proximal jejunum, Muc2 was upregulated and Muc5ac downregulated (Amat et al., 2017). *In vitro* alterations to mucin gene expression were found to be *Giardia* cysteine protease dependent (Amat et al., 2017; Fekete et al., 2019). The presence of a functional mucus layer also protects against parasitic colonization, as in MUC2 deficient mice, which lack a functional mucus gel barrier in the small and large intestines, *Giardia* burden is significantly elevated compared to wild-type mice (Amat et al., 2017).

Although the cause-to-effect relationship between dysbiosis and mucosal disruptions in the context of *Giardia* infections still requires further investigation, reciprocal relationships likely exist, wherein the disruption of mucus drives bacterial dysbiosis and vice versa. *Giardia*-driven dysbiosis may result in changes to mucus properties, including glycosylation patterns and penetrability. Changes to populations of mucolytic bacteria may result in altered mucus degradation throughout the gut, even in regions of the gut, like the large intestine, where *Giardia* does not colonize but still disrupts the mucus layer (Amat et al., 2017). Indeed, an increase in *Akkermansia* has been demonstrated in *G. duodenalis* GS/M infected mice (Amat et al., 2017). Therefore, we can hypothesize that altered production and secretion of mucus throughout the gut may also be driven by bacteria or bacterial metabolites. Individual variations in mucus structure and composition as a result of individual microbiome variability may impact susceptibility to *Giardia* colonization and the severity of symptoms. These effects may persist long after parasite clearance, potentially contributing, at least in part, to development of post-infectious disorders in humans.

### Probiotics: The *Giardia* Kryptonite

Probiotics, which are defined as “live microorganisms that, when administered in adequate amounts, confer a health benefit to the host,” represent a large class of microorganisms, including bacteria and yeast, that may have protective effects against parasitic infection or other GI diseases (Hill et al., 2014). Probiotic strains must meet international food safety agencies criteria for consumption, such as “Generally Recognized as Safe” status (Food and Drug administration criteria) or “Qualified Presumption of Safety” status (European Food Safety Authority; Sorokulova, 2008; Alazzaz, 2020). The largest bacterial group of probiotic strain candidates is lactic acid bacteria, mainly represented by the genus *Lactobacillus*, which are characterized by the production of lactic acid as an end product of glucose metabolism. Other common and well-characterized probiotic strains belong to other genera such as *Bifidobacterium*, *Enterococcus*, and *Saccharomyces* (yeast). Following oral administration, probiotic microbes must survive gastric acids and bile acid toxicity, compete with the gut commensal microbiota for ecological niches, and adhere to the mucosa in the small intestine (Figueroa-Gonzalez et al., 2011; Markowiak and Slizewska, 2017). In most cases, probiotic strains are screened for pathogen inhibition, either *via* secretion of bacteriocins, competitive advantage for nutrients, or by stimulating the host immune response against pathogens (Figueroa-Gonzalez et al., 2011; Markowiak and Slizewska, 2017).

Over the past 20 years, numerous studies have explored the anti-*Giardia* properties of probiotic strains as an alternative strategy for prevention and treatment of giardiasis. In particular, lactobacilli are a good candidate since they share the same ecological niches as *Giardia* trophozoites in the upper small intestine. *In vitro* and *in vivo* studies showed that several lactobacilli species can exert cytostatic and/or cytotoxic effects against *Giardia* trophozoites (Table 1). Other probiotic strains from other genus or species showed potent anti-*Giardia* properties *ex-vivo* and *in vivo*, such as *Bifidobacterium* spp., *Weissella paramesenteroides*, *Enterococcus faecium*, *Saccharomyces boulardii*, as well as multi-strain probiotic and mixed bacteria and yeast cultures such as kefir grains (composed of *Lactobacillus* spp., *Lactococcus lactis Leuconostoc mesenteroides*, *Saccharomyces* spp., and *Acetobacter* spp.) and Slab51 (*Lactobacillus* spp., *Streptococcus thermophilus*, and *Bifidobacterium* spp.; Table 1).

**Table 1:** Probiotic strains exerting anti-*Giardia* activities *in vitro*, *ex vivo*, and *in vivo*.

	*Giardia* isolate	Anti-*Giardia* activity *in vitro*	Anti-Giardia activity *in vivo* (reduced parasitic load)	Animal model	References
*Bifidobacterium longum* 5(1A)	*Giardia duodenalis strain* GS/M clone H7	N/A	Yes	4–6 weeks Mongolian gerbils	Fonseca et al., 2019
*Enterococcus faecium* SF68	*Giardia duodenalis strain* GS/M clone H7	N/A	Yes	6 weeks C57BL/6 mice	Benyacoub et al., 2005
*Enterococcus faecium* SF68	Unknown	N/A	Yes (combined with metronidazole treatment)	16 weeks to 3 years dogs	Fenimore et al., 2017
*Lactobacillus brevis* CNRZ 1845	*Giardia duodenalis* strain WB	Yes	N/A	N/A	Allain et al., 2018a
*Lactobacillus casei* MTCC 1423	*Giardia duodenalis* strain Portland-I	N/A	Yes	5–6 weeks BALB/c mice	Shukla et al., 2008; Shukla and Sidhu, 2011; Shukla et al., 2013
*Lactobacillus casei* ATCC 393	*Giardia duodenalis* strain WB	Yes	N/A	N/A	Allain et al., 2018a
*Lactobacillus crispatus* CIP 103606	*Giardia duodenalis* strain WB	Yes	N/A	N/A	Allain et al., 2018a
*Lactobacillus gasseri* CNCM I-4884	*Giardia duodenalis* strain WB	Yes	Yes	OF-1 Suckling mice	Allain et al., 2018a
*Lactobacillus gasseri* ATCC 3323	*Giardia duodenalis* strain WB	Yes	N/A	N/A	Allain et al., 2018a
*Lactobacillus helveticus* ATCC 11977	*Giardia duodenalis* strain WB	Yes	N/A	N/A	Allain et al., 2018a
*Lactobacillus johnsonii* La1,(NCC533)	*Giardia duodenalis* strain WB	Yes	Yes	Mongolian gerbils (young adults)	Perez et al., 2001; Humen et al.,2005
*Lactobacillus johnsonii* La1,(NCC533)	*Giardia duodenalis* strain WB	Yes	No	OF-1 Suckling mice	Allain et al., 2018a
*Lactobacillus johnsonii* CIP, *Lactobacillus johnsonii* 103653, *Lactobacillus johnsonii* CIP 103614, *Lactobacillus johnsonii* CIP 103654, *Lactobacillus johnsonii* CIP 103781, *Lactobacillus johnsonii* CIP 103652, *Lactobacillus johnsonii* CIP 103786, *Lactobacillus johnsonii* ATCC 33200, *Lactobacillus johnsonii* CNRZ 218, and *Lactobacillus johnsonii* CNRZ 1897	*Giardia duodenalis* strain WB	Yes	N/A	N/A	Allain et al., 2018a
*Lactobacillus reuteri* CNRZ 431	*Giardia duodenalis* strain WB	Yes	N/A	N/A	Allain et al., 2018a
*Lactobacillus rhamnosus* GG	*Giardia duodenalis* strain Portland-I	N/A	Yes	5–6 weeks BALB/c mice	Goyal et al., 2011, 2013; Goyal and Shukla, 2013
*Saccharomyces boulardii*	Unknown	N/A	Yes (combined with metronidazole treatment)	Humans (clinical study)	Besirbellioglu et al., 2006
*Saccharomyces boulardii*	*Giardia duodenalis strain* BHRA-93	N/A	Yes	4–6 weeks Mongolian gerbils	Ribeiro et al., 2018
*Weissella paramesenteroides* WpK4	*Giardia duodenalis strain* GS/M clone H7	N/A	Yes	4–6 weeks Mongolian gerbils	Fonseca et al., 2019
kefir grains CIDCA AGK1^1^	*Giardia duodenalis strain* GS/M clone H7	N/A	Yes	4–5 weeks C57BL/6 mice	Franco et al., 2013
Slab51^2^	*Giardia duodenalis* strain WB	N/A	Yes (*ex vivo* anti-*Giardia* activity)	Duodenum sections of CD-1(ICR)BR mice	Perrucci et al., 2019

1 CIDCA AGK1: *Lactobacillus plantarum*, *Lactobacillus kefir*, *Lactococcus lactis* subsp. lactis, *Leuconostoc mesenteroides*, *Saccharomyces* spp., and *Acetobacter* spp.

2 Slab51: *Streptococcus thermophilus* DSM 32245, *Bifidobacterium lactis* DSM 32246, *Bifidobacterium lactis* DSM 32247, *Lactobacillus acidophilus* DSM 32241, *Lactobacillus helveticus* DSM 32242, *Lactobacillus paracasei* DSM 32243, *Lactobacillus plantarum* DSM 32244, and *Lactobacillus brevis* DSM 27961.

The mechanisms underlying these inhibitory effects remain unclear. Probiotic strains may act like a “Swiss Army Knife” against enteropathogens by secreting anti-microbial factors, competing for ecological niches, enhancing the host innate and adaptive immune response, regulating intestinal barrier integrity, and promoting mucosal healing (Taverniti and Guglielmetti, 2011; Travers et al., 2011). *In vivo* models of giardiasis revealed that the probiotic supplementation was associated with potent immuno-modulatory effects, with upregulation of anti-inflammatory cytokines, such as IL-10, or increases in *Giardia*-specific IgA and IgG (Benyacoub et al., 2005; Shukla and Sidhu, 2011). The ability of probiotics to compete for ecological niches is another important aspect of their anti-*Giardia* effects, as *Giardia* must resist the hostile microenvironmental conditions of the upper small intestine, where bile and digestive enzymes pour in. For instance, lactobacilli’s bile salt hydrolase (BSH) enzymatic activity, a key bacterial cellular process for bile detoxification, is associated with the release and accumulation of deconjugated bile salts, which are toxic for *Giardia* trophozoites (Travers et al., 2016). Indeed, a recent report showed that the BSH activity of lactobacilli strains is positively correlated with their anti-*Giardia* effects (Allain et al., 2018a). Further investigations showed that recombinant BSHs from the probiotic strain *L. johnsonii* La1 can directly inhibit the growth of *Giardia* trophozoites *in vitro* and *in vivo* (Allain et al., 2017). Other reports suggest that lactobacilli, such as *L. acidophilus* P106, secrete bacteriocins that exert direct cytotoxic effects on *Giardia* trophozoites (Amer et al., 2014). Consumption of key metabolites, such as L-arginine, nucleosides, purines, and pyrimidines, by probiotic strains can also create a competitive advantage over trophozoites in the small intestine (Lindmark and Jarroll, 1982; Jarroll et al., 1989; Baum et al., 1993; Jarroll, 2011; Allain et al., 2018b). Finally, other probiotic properties must be considered, such as the presence of adhesion factors and competition for binding sites on enterocytes *via* carbohydrate-binding specificities (Pique et al., 2019). More clinical studies are needed to establish the benefits of probiotics in giardiasis, as well as in other disorders of the gut.

## Alterations To The Microbiota During *Giardia* Infection

### Alterations to the Microbiome in Clinical Studies of Giardiasis

**Figure 1:**
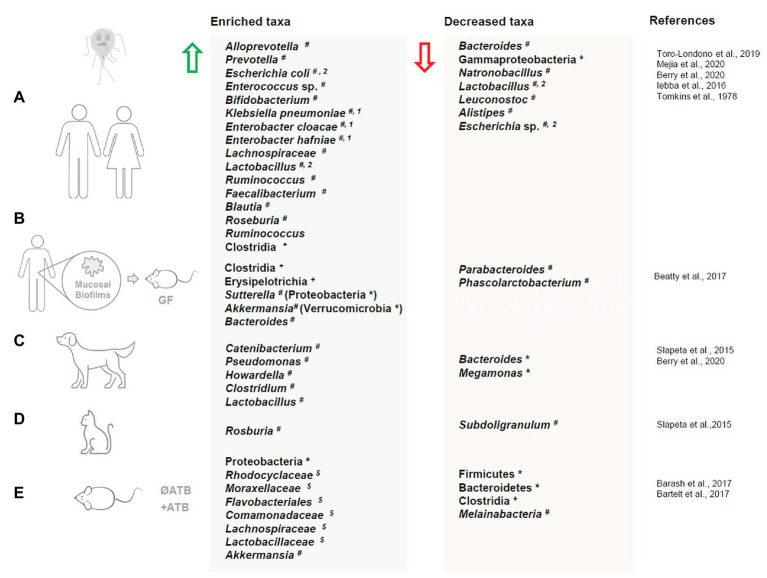
Dysbiosis in humans and animals upon *Giardia duodenalis* infection. Changes in gut microbiota taxa composition upon *Giardia duodenalis* infection in live hosts: **(A)** human, **(B)** gut microbiota biofilms isolated from human biopsies exposed to *Giardia* and administered to germ free mice, **(C)** dogs, **(D)** cats, and **(E)** mouse. Enriched taxa are indicated by a green arrow. Decreased taxa are indicated by a red arrow. ATB = antibiotic treatment; * = phylum; + = class; $ = family # = genus; 1 = patients with severe malabsorption; 2 = taxa both enriched and decreased in separate studies. Microbiota dysbiosis in response to *Giardia muris* infections (i.e., Yordanova et al., 2019) is not represented in this figure.

Several recent studies have begun to shed light on bacterial taxonomic shifts associated with *Giardia* infections in humans (Figure 1). In a study of individuals from sub-Saharan Africa, microbial taxonomic differences were assessed in males and females ranging from 1 to 74 years of age infected with *Giardia* and other protozoa. Temporal temperature gradient gel electrophoresis (TTGE) analysis revealed that individuals infected by *Giardia* alone have a distinct TTGE profile compared with uninfected individuals or those infected with *Entamoeba* or *Blastocystis* (Iebba et al., 2016). Dispersion matrices showed that *Giardia* infection was positively correlated with an increase in *Escherichia coli*, *Enterococcus* species, and bifidobacteria. The authors failed to establish a correlation between *Giardia* infection and a dysbiosis index, which characterized by the ratio between the relative abundances of *Faecalibacterium prausnitzii* and *E. coli* (Iebba et al., 2016). An early study of patients with *Giardia* predicted that the small intestinal microbiota composition can influence the pathogenesis and clinical manifestations of giardiasis. Increased jejunal concentrations of enterobacteria (i.e., live bacteria isolated from mucosa and luminal fluids) such as *Klebsiella pneumoniae*, *Enterobacter cloacae*, and *Enterobacter hafniae* were observed only in patients with severe intestinal malabsorption, while enterobacteria overgrowth was not observed in patients with mild malabsorption (Tomkins et al., 1978). In an attempt to investigate the role of microbiota-*Giardia* interactions in the pathogenesis of giardiasis, 31 facultative and strictly anaerobic duodenal bacteria isolated from patients with symptomatic giardiasis were administered in different combinations to germ-free mice. While bacteria cocktails alone failed to mimic *Giardia* pathogenesis, co-administration with *G. duodenalis* isolate BT6 resulted in an increase in *Giardia*-specific IgA, IgM, and IgG levels, suggesting that the small intestine microbiota can enhance the host immune response to *Giardia* infection (Torres et al., 2000).

16S rRNA gene sequencing analysis of fecal microbiota from Colombian children infected with one or several parasites revealed that individuals infected with *Giardia* only (*n* = 5) had distinct microbial signatures compared to children with no detectable gut parasites. The *Giardia* group had a lower Shannon’s alpha diversity index and lower species richness compared to uninfected controls (Toro-Londono et al., 2019). Despite recent doubts about the robustness of “enterotypes,” it has been suggested that gut bacterial species may be clustered into distinct categories: (1) enterotype I, dominated by *Bacteroides*; (2) enterotype II, dominated by *Prevotella*; and (3) enterotype III, dominated by *Ruminococcus*. These may be influenced by dietary habits and environment (Rinninella et al., 2019). In this study, children infected with *Giardia* present a shift from enterotype I to enterotype II, characterized by an increase of *Prevotella* and a decrease of *Bacteroides* (Toro-Londono et al., 2019). Significant increases in *Prevotella* were also observed in Argentinian children infected with *Giardia* compared to uninfected individuals (Mejia et al., 2020). Interestingly, *Giardia* infection was associated with a decreased abundance of microbiome Vitamin B12 (cobalamin) biosynthesis genes (CbiM), which has been associated with impaired growth and development in children (Mejia et al., 2020).

Recently, a study employed a database mining approach to examine data from the Global Enteric Multicenter Study (GEMS) and “Malnutrition and Enteric Disease Study” (MAL-ED). Human cohorts from four countries were studied to determine whether *Giardia* infection was associated with a distinct microbiota signature and/or microbiota dysbiosis in young individuals. Among the GEMS participants, 215 individuals tested positive for *Giardia* and were subclassified as having either moderate to severe diarrhea (MSD) or no MSD. Metagenomic biomarker discovery analysis (LEfSe score) revealed that *Giardia* infection was associated with an enrichment of *Prevotella* and a reduction in Gammaproteobacteria, regardless of the presence of mild to severe diarrhea (Berry et al., 2020). To a lesser extent, *Giardia* infection was also associated with an increase of *Lachnospiraceae*, *Ruminococcus*, and *Clostridiales*. Conversely, *Giardia* infection was correlated with a decrease of *Natronobacillus*, *Lactobacillus*, and *Leuconostoc*. Similar results were observed in MAL-ED participants (Peru), where *Giardia* Infection was associated with an enrichment of *Prevotella* and a reduction in *Escherichia* (Gammaproteobacteria; Berry et al., 2020). LEfSe also revealed an association between *Giardia* infection and an increase of *Faecalibacterium*, *Blautia*, *Roseburia*, *Ruminococcus*, and *Ruminoccaceae*, and an increase of *Lachnospiraceae* and *Lactobacillus* contrary to the GEMS study. Altogether these multicentered findings suggest that *Giardia* colonization is associated with a robust increase in *Prevotella* (enterotype II). Interestingly, *Prevotella* has previously been found to promote differentiation of Th17 cells (Calcinotto et al., 2018), which play a critical role in control of *Giardia* infection.

### Alterations to the Microbiome in Murine Models of *Giardia* Infection

*Giardia* infection is associated with an altered gut microbiota composition throughout the gastrointestinal tract in mice (Figure 1.). A recent study showed that the microbial composition is altered at the phylum level (16S rRNA gene sequencing) upon infection with *G. duodenalis* isolate GS/M in both the small and large intestines, with a general increase in Proteobacteria and decrease in Firmicutes diversity in both antibiotic treated and antibiotic naive mice (Barash et al., 2017). In both antibiotic treated and antibiotic naive mice, significant decreases in Clostridia, a strict anaerobe, were observed throughout the small and large intestines. Strict aerobes including *Rhodocyclaceae*, *Moraxellaceae*, *Flavobacteriales*, and *Comamonadaceae* were enriched in the large and small intestines. *Melainabacteria* was found to be reduced in the proximal small intestines of antibiotic treated mice, but enriched in the distal small intestine. In contrast, in antibiotic naive mice *Melainabacteria* was reduced in both the proximal and distal small intestine. Taxa that were found to be enriched are generally considered to be metabolically flexible, and so will thrive under stress conditions induced by the presence of the pathogen, including arginine deprivation and altered nutrient availability (Barash et al., 2017). In a second study of *G. duodenalis* strain H3 infected mice, 16S rRNA gene sequencing revealed a decrease in Bacteroidetes was observed in the upper small intestine while Firmicutes abundance remained relatively consistent compared to uninfected mice (Bartelt et al., 2017). Interestingly, a slight increase in OTUs for the mucolytic species *Akkermansia* was also observed (Bartelt et al., 2017). In a recent study using *G. duodenalis* strain GS/M (clone H7), greater microbiome shifts were observed in infected adult mice than in acutely infected neonates using 16S sequencing. This was attributed both to the influence of mature vs. immature immune systems and to *Giardia*-induced enhancement of bile secretion in adult mice (Riba et al., 2020). Indeed, populations of the bile salt deconjugating species *Enterorhabdus* were increased (Riba et al., 2020). In the large intestines, there was an overall increase in OTUs, and while the abundance of *Staphylococcus* decreased, increased populations of *Coriobacteriaceae* were observed (Riba et al., 2020).

Microbiota composition has also been investigated in immunocompromised animals (see section Microbiota-Immune System Interactions During *Giardia* Infection). In both IL-10^−/−^ and Tcra^−/−^ mice (animals deficient in alpha beta T cell receptor and devoid of CD4+ and CD8- and CD4− and CD8+ T cells), *Giardia* infection led to a significant reduction of microbial diversity (16S rRNA sequencing) that was not observed in wild-type mice. A reduction of Bacteroides and Firmicutes and an increase in Proteobacteria was evident, suggesting that regardless of different genetic backgrounds, mice with *Giardia* infections tend to develop microbiota dysbiosis (Dann et al., 2018). Expansion of Proteobacteria has previously been associated with intestinal inflammation and correlates with a disruption of the anaerobic state of the healthy colon (Litvak et al., 2017).

### Influence of Host Nutritional Status on *Giardia*-Microbiome Interactions

Diet and the host nutritional status are known to play a key role during parasitic infections (Shea-Donohue et al., 2017). In giardiasis, micronutrient deficiencies, such as zinc, iron, and vitamin A, are associated with increased host susceptibility to infection, impaired immune responses and intestinal malabsorption in young individuals (Duncombe et al., 1980; Astiazaran-Garcia et al., 2010, 2015; Quihui-Cota et al., 2012; Bartelt et al., 2013; Inigo-Figueroa et al., 2013). Supplementation of micronutrients and trace elements in children led to a reduction of the frequency and severity of diarrhea and a decrease in infection rate, indicating that diet intervention may be considered as an efficient therapeutic strategy (Lima et al., 2010; Astiazaran-Garcia et al., 2015). In mice, dietary fibers, dietary fat, protein deficiencies, and prebiotic fibers can modulate the outcome of giardiasis (by modulating parasite persistence, host immunity, intestinal barrier dysfunction, etc.); however, few studies have linked those effects to the gut microbiota (Upadhyay et al., 1985; Hamadto et al., 1989; Leitch et al., 1989; Magne et al., 1994; Rodrigues et al., 1994; Diaz-Cinco et al., 2002; Suh et al., 2004; Erlandsen, 2005; Gomes et al., 2012; Bartelt et al., 2013; Ventura et al., 2013; Shukla et al., 2016; Burgess et al., 2019). Recent evidence suggests that diet composition may at least in part modulate the microbiota dysbiosis associated with giardiasis. In mice fed a protein deficient diet (PD), both trophozoite burden and persistence have been shown to be increased compared to mice fed an isocaloric conventional diet (CD). Protein deficiency, in the absence of infection, causes significant dysbiosis in mice, as well as elevated bacterial abundance in the duodenum (Bartelt et al., 2017). Similarly, in *Giardia*-infected mice, distinct microbiome profiles are observed when comparing infected mice fed PD and CD. Greater duodenal bacterial abundance was observed *via* 16S rRNA sequencing in *G. duodenalis* H3 infected PD mice compared to infected CD mice. Mice fed PD diets showed increased Firmicutes: Bacteroidetes ratios, with a significant increase in the abundance of *Clostridiales*. *Giardia* infection tended to further enhance Firmicutes abundance, in addition to reducing Bacteroidetes abundance (Bartelt et al., 2017). *Giardia*-induced growth impairment was enhanced by the PD diet and could be ameliorated by antibiotic treatment, suggesting an important role for the microbiota in the cooperative effects of *Giardia* infection and protein deficiency in growth impairment (Bartelt et al., 2017).

### Alterations to the Microbiome in *Giardia*-Infected Companion Animals

Companion animals are frequent carriers of both symptomatic and asymptomatic *Giardia* infections (Figure 1; Tysnes et al., 2014; Bouzid et al., 2015; Stafford et al., 2020). The prevalence of giardiasis varies widely by region but has been estimated *via* meta-analysis of prevalence reports to be approximately 15% in dogs and 12% in cats (Bouzid et al., 2015). To date, only a handful of studies have characterized the gut microbiota of these infected animals. In free-roaming dogs, *Giardia* infection was associated with significant alteration of the fecal microbiome at the genus level, as analyzed through 16S sequencing. *Giardia* positive groups showed an increase in *Catenibacterium*, *Pseudomonas*, and *Howardella*, while *Giardia* negative groups had higher levels of Bacteroides and *Pseudobutyrivibrio* (Slapeta et al., 2015). Domesticated dogs infected with *Giardia* in the United States similarly showed significant changes to fecal microbiome beta-diversity compared to uninfected animals (Berry et al., 2020). Of all the parasites studied (*Giardia*, Cystoisospora, hookworm, whipworm, ascaris, and tapeworm), *Giardia* alone, and *Giardia* with polymicrobial infections, caused the most significant difference in the beta diversity as compared to non-infected control animals. Specifically, *Giardia* was associated with an enrichment of *Clostridium* and *Lactobacillus* and a reduction in Bacteroides as determined by both Bray-Curtis and weighted UniFrac metrics, even when the confounding variables such as age and sex were controlled for. The distinct microbiome profile associated with *Giardia* positive samples was evident even in the context of co-infections and was observed with and without a history of antibiotic treatment or diarrheal symptoms (Berry et al., 2020). In fact, the *Giardia* co-infected animals were indistinguishable in terms of beta diversity from the animals that were infected with *Giardia* alone.

In Felidae, *Giardia*-infected cats, significant differences in overall microbiome profiles have yet to be documented; however, several genera appear to be altered in *Giardia*-positive cats, with increased *Rosburia* and decreased *Subdoligranulum* compared to *Giardia*-negative animals. In addition, the overall number of operational taxonomic units (OTUs) may be increased in *Giardia* infected cats (Slapeta et al., 2015).

### *Giardia* Trophozoites and Secretory-Excretory Products Promote Formation of Pathobionts

As described above (section Structure and Composition of the Intestinal Microbiota), gut microbes can aggregate as a poly-microbial structure known as a biofilm, which (i) facilitates adhesion to their substrate, (ii) helps them to withstand shear forces (shear forces range from 0.02 to 35 dynes/cm^2^ in force value in the gut) and (iii) increases their persistence and resistance in the gut through production of an extracellular matrix (ECM; Avvisato et al., 2007; Trujillo-de Santiago et al., 2018; Buret et al., 2019). To characterize the effects of *Giardia* on gut microbiota biofilms, a recent study examined interactions between *G. duodenalis* isolate NF and multi-species biofilms cultured from healthy human intestinal biopsies using the Calgary Biofilm Device (Beatty et al., 2017). Scanning electron and representative confocal scanning laser micrographs revealed that *Giardia* excretory/secretory products (ESPs) were able to actively degrade the ECM of human microbiota biofilms in a cysteine protease dependent manner, promoting increased dispersal of planktonic bacteria from the biofilm. When exposed to the intestinal epithelial cell line Caco-2, these dispersed planktonic bacteria demonstrated increased virulence compared to dispersed bacteria from control biofilms that had not been exposed to *Giardia*. These *Giardia*-induced pathobionts promoted the release of pro-inflammatory CXCL-8, in association with an upregulation of TLR4 pathway signaling (Beatty et al., 2017). Moreover, these bacteria degraded tight junctional proteins, such as ZO-1, and induced epithelial apoptosis, which together can promote bacterial translocation by reducing epithelial barrier function. To further characterize the effects of *Giardia* on microbiota biofilms, germ-free C57Bl/6 mice were reconstituted with biofilm-dispersed planktonic bacteria obtained from *Giardia*-exposed or control biofilms. Histological examination revealed that *Giardia*-exposed bacteria-induced lymphocyte aggregation in the colonic mucosa and upregulated the pro-inflammatory cytokines IL-1*β*, TNF-*α*, IFN-*γ*, and IL-17 in colonic tissues 14 days post challenge compared with mice given control bacteria (Beatty et al., 2017). Mouse fecal microbiota composition (16s rRNA gene sequencing) in germ-free mice given *Giardia*-treated biofilm bacteria compared to those given untreated biofilm bacteria showed an overall increase in Firmicutes (*Clostridiale*s) and Bacteroidetes at the phylum level. At the genus level, there was an increase of *Sutterella*, which is associated with interleukin secretion and colitis in humans, and a decrease of *Parabacteroides* and *Phascolarctobacterium* (Figure 1; Hiippala et al., 2016). Together, the data reveal a novel mechanism by which exposure to an enteropathogen, *Giardia* in this case, may drive pathogenesis by converting commensal biofilm microbes into pathobionts.

The ability of *Giardia* trophozoites to promote the release of pathobionts and alter bacterial virulence was further assessed *in vivo* using the nematode *Caenorhabditis elegans* as a model (Gerbaba et al., 2015, 2017). Alone, neither non-pathogenic *E. coli* strains OP50 or HB101 nor *G. duodenalis* isolate NF trophozoites had an effect on *C. elegans via*bility. However, exposure to *E. coli* strain OP50 that had been grown in co-culture with *G. duodenalis* trophozoites or its ESPs resulted in the death of the nematode within 24 h. Similar observations were made in *C. elegans* exposed to *E. coli* HB101 co-cultured with *G. duodenalis* (Gerbaba et al., 2015). Synergistic lethal effects of *Giardia* and *E. coli* were associated with facilitation of bacterial colonization of the *C. elegans* intestine. Transcriptomic analysis conducted in this study revealed that *Giardia* ESPs altered the expression of over 100 genes in *E. coli* HB101, including virulence factor genes, suggesting that *Giardia*-induced functional changes in commensal species can increase their toxicity to the host (Gerbaba et al., 2015). Additional experiments were conducted on human microbiota biofilms obtained from either healthy donors or from inflamed sites in patients with ulcerative colitis (UC). Upon prior exposure to *G. duodenalis*, both microbiota from healthy donors and UC patients became lethal toward *C. elegans*, while microbiota not exposed to *Giardia* showed little to no toxicity. Altogether, these findings indicate that *Giardia* can actively disrupt human commensal microbial biofilms and promote the release of pathobionts in the gut that may exert further pro-inflammatory effects. These effects may contribute at least in part to acute and post-infectious disease in giardiasis.

Additionally, recent studies have also elucidated that *Giardia* can release extracellular vesicles (EVs) that contain well-characterized virulence factors (Evans-Osses et al., 2015; Moyano et al., 2019; Gavinho et al., 2020). These EVs have been shown to play an important role in host-*Giardia* interaction. Whether *Giardia* EVs represent a potential mode of interaction between the parasite and the host’s microbiota warrants further investigation.

## Giardiasis in Co-Infections: A Double-Edged Sword

### Prevalence of Enteropathogens in Concomitant Infection With *Giardia* in Humans

Polymicrobial infections are defined as the presence of microorganisms that, under certain circumstances, can establish a concomitant infection (Brogden et al., 2005). These infections, mostly transmitted *via* the fecal-oral route, are very common in developing countries with poor sanitation and represent a burden on the development of children (Ryan and Caccio, 2013; Muhsen et al., 2014). Infection with *G. duodenalis* has been associated with concomitant infections with a variety of gut bacteria, viruses, and parasites. For example, in Venezuelan children, 62% with mild to moderate *Ascaris lumbricoides* infection were co-infected with *G. duodenalis*, and 45% of children with low *A. lumbricoides* burden were also infected with *Giardia* (Hagel et al., 2011). During an outbreak of cryptosporidiosis in a hospital ward in China, 6.3% of the patients infected with *Cryptosporidium* were also positive for *G. duodenalis* (Wang et al., 2013). Additionally, there has been a case of co-infection of *G. duodenalis* and *Cyclospora cayetanensis* in an immunocompetent patient with prolonged diarrhea in Turkey (Koru et al., 2006). Concomitant infection with *C. difficile* and *G. duodenalis* has also been reported (Khatami et al., 2004). In France, 4 of 25 patients diagnosed with Whipple disease were also infected with *Giardia* (Fenollar et al., 2003). In India, 32 and 19% of the diarrheal patients infected with *Giardia* were also coinfected with *Vibrio cholera* and rotavirus, respectively (Mukherjee et al., 2014). Concomitant infection of *Giardia* with *E. coli*, *Campylobacter* spp., *Salmonella* spp., and rotavirus are also frequent (Bilenko et al., 2004). *Giardia* has also been detected frequently in non-diarrhoeal stools along with *Campylobacter* spp., enteroaggregative *E. coli*, norovirus, LT-producing enterotoxigenic *E coli* (EPEC), and typical and atypical enteropathogenic *E. coli* strains (Platts-Mills et al., 2015). In Nicaragua, 70% of *G. duodenalis* cases were co-infected with another enteropathogen, most commonly norovirus, sapovirus, or EPEC (Becker-Dreps et al., 2014). A recent report indicated that in a MAL-ED cohort, 14 of 215 *Giardia* infected children were also coinfected with either *Cryptosporidum* or rotavirus (Berry et al., 2020).

Several reports suggest that concomitant infections with *G. duodenalis* and *Helicobacter pylori* are frequent. In Ugandan children between 0 and 12 years of age, the incidence of *Giardia* infection was 3-fold higher (odds ratio of 2.9) in the presence of *H. pylori* compared to single-species infections (Ankarklev et al., 2012). In Egyptian children with recurrent abdominal pain (RAP), concomitant infection with *H. pylori* and *Giardia* was found in 40% of RAP patients compared to 12.2% in controls (Eldash et al., 2013). Similarly, prevalence of concomitant *H. pylori* infection and *Giardia* was 22.4% in RAP patients in Turkey, compared to 6.8% in the control groups (Zeyrek et al., 2008). In Italy, *H. pylori* was present in 37 of the 41 patients with gastric giardiasis (Doglioni et al., 1992). In Brazilian children, seropositivity to *H. pylori* antibodies was associated with the presence of *Giardia* with an odds ratio of 3.2 (Moreira et al., 2005). Gastric biopsy examination of patients with chronic cholecystitis associated with chronic gastroduodenitis revealed a high prevalence of co-infection with *H. pylori* and *G. duodenalis* (Isaeva and Efimova, 2010). Whether and how polyparasitism in giardiasis may contribute to disease is the topic of intense research activities.

### *Giardia* and Enteroaggregative *Escherichia coli* Synergize to Enhance Malnutrition and Growth Impairment by Inducing Metabolic Dysfunctions and Immune Perturbations

In certain cases, co-infections with *Giardia* and other pathogens can result in negative outcomes for the host. Enteroaggregative *Escherichia coli* (EAEC) is one of the most common enteropathogens isolated from malnourished children, and as such is commonly found in *Giardia* co-infections (Platts-Mills et al., 2015). Using a murine model of protein malnutrition, recent findings observed that coinfection with *G. duodenalis* and EAEC promotes growth impairment, microbiota-dependent delayed parasite clearance, microbial metabolic perturbations in the gut, and an alteration of local host immune responses against EAEC (Bartelt et al., 2017). Combination of the two pathogens resulted in enhanced weight loss in mice fed a protein deficient (PD) diet compared to uninfected controls, whereas in mice fed with a conventional diet (CD), the infected groups showed no significant weight loss. These data provide compelling evidence that, in malnourished hosts, *Giardia* infection and polymicrobial infections may synergize to exacerbate stunted growth. In addition, in mice co-infected with EAEC, *Giardia* infection may cause extraintestinal manifestations by mediating and attenuating the cytokine response of bone marrow-derived dendritic cells (Burgess et al., 2019). A previous report had demonstrated growth impairment and decreased inflammatory responses in malnourished mice infected with *Giardia* alone (Bartelt et al., 2013). Together, these results are in accordance with epidemiological data showing that *Giardia* infection is more likely to contribute to decreased immune functions and growth impairment in malnourished children (Fink and Singer, 2017).

### Immunomodulation by *Giardia* Attenuates Enteropathogen-Induced Inflammation

Evidence from epidemiological studies indicate that co-infections involving *G. duodenalis* may be associated with a protective effect against enteropathogen-driven diarrhea in developing countries (Bilenko et al., 2004; Veenemans et al., 2011; Muhsen et al., 2014; Cotton et al., 2015). An example of this protective effect comes from studies conducted on Bangladeshi and Tanzanian children, whereby children infected with *Giardia* present with reduced diarrheal illness as well as reduced serum inflammatory scores (Bilenko et al., 2004; Haque, 2007; Veenemans et al., 2011; Muhsen et al., 2014).

In an attempt to explain the mechanisms underlying the protective effects of *Giardia* against attaching and effacing pathogens (A/E pathogens), a murine model of coinfection with *Giardia muris* and the mouse enteropathogen *Citrobacter rodentium* was established (Manko et al., 2017). Results showed that *G. muris* can reduce the symptoms of *C. rodentium*-induced colitis, including weight loss, intestinal permeability, and histopathological damages, by enhancing, at least in part, the production of mucosal antimicrobial peptides (AMPs) such as Mouse *β*-defensin 3 and Trefoil factor 3 (TFF3; Manko et al., 2017). Similarly, coinfection with *G. duodenalis* isolate NF and enteropathogenic *E. coli* (EPEC) of human intestinal epithelial cells results in cathepsin B-dependent increases in human β-defensin 2 and TFF3 gene expression, two major AMPs produced by intestinal epithelia (Manko et al., 2017). Elevated levels of MMP7, an intestinal matrix metalloprotease involved in *α*-defensin production by Paneth cells, have been previously observed in response to *Giardia* infection in mice (Tako et al., 2013). In addition, *Giardia* trophozoites exert anti- EPEC effects *ex-vivo* in crypt killing assays (Manko et al., 2017). Recent research also explored the role of the NLRP3 inflammasome in the protective effects of *Giardia* during co-infections. The NLRP3 inflammasome is activated by other protozoan parasites such as *Leishmania* spp., *Plasmodium* spp., *Entamoeba* spp., *Naegleria* spp., *Trypanosoma cruzi*, and *Toxoplasma gondii* (Mortimer et al., 2014, 2015; Kim et al., 2016; de Carvalho et al., 2020). New findings now demonstrate that upregulation of mucosal AMPs and reduction of *C. rodentium*-induced colitis in mice co-infected with *Giardia* and *C. rodentium* is mediated by the activation of the NLRP3 inflammasome pathway (Manko-Prykhoda et al., 2020). In human intestinal epithelial cell lines, chemical inhibition of the NLRP3 inflammasome blocked the upregulation of AMP gene expression. Moreover, the antimicrobial activity of colonic crypt cells against A/E enteropathogens is enhanced by *Giardia* in a NLRP3-dependent manner (Manko-Prykhoda et al., 2020). The data uncover a novel protective role for NLRP3 during polyparasitism.

Other studies also investigated the immunomodulatory properties of *Giardia* against inflammatory responses to *Clostridium difficile* or *Salmonella* spp., two enteropathogens found concomitantly with *Giardia* (Bilenko et al., 2004; Khatami et al., 2004). In an *in vivo* model of *C. difficile* toxin A and B-induced colitis, *G. duodenalis* was shown to dampen granulocyte infiltration, decrease colonic myeloperoxidase activity (a marker of inflammation) and attenuate the expression and secretion of pro-inflammatory mediators and neutrophil chemo-attractants, in a *Giardia* isolate-dependent manner (Cotton et al., 2014b). In intestinal epithelial cell cultures stimulated with *Salmonella enterica* (serovar *Typhimurium*), *Giardia* can actively degrade CXCL-8, a potent pro-inflammatory cytokine and neutrophil chemoattractant, in a cysteine protease-dependent fashion (Cotton et al., 2014a). Consistent with these observations, recent reports demonstrated that *Giardia* cysteine proteases can degrade immunoglobulins, chemokines and other cytokines, tight junctional proteins, and epithelial cytoskeletal villin (Bhargava et al., 2015; Liu et al., 2018, 2019; Ortega-Pierres et al., 2018; Allain et al., 2019). Together, these data further support the hypothesis that *Giardia* modulates the host response to enteropathogens during co-infections.

## Post-Giardiasis Intestinal Disorders: A Role For Bacterial Translocation

Irritable bowel syndrome (IBS) is a functional gastrointestinal disorder, characterized by recurring or chronic gastrointestinal symptoms including abdominal discomfort, altered gut motility, and visceral hypersensitivity (Simsek, 2011). One of the subtypes of IBS, post infectious irritable bowel syndrome (PI-IBS), is characterized by the manifestation of IBS symptoms following infections by enteric pathogens (Heitkemper et al., 2011). Recent research has demonstrated that *Giardia* infection can lead to the development of gastrointestinal disorders even after the successful clearance of the parasite. Indeed, *Giardia* infection can increase the risk of developing PI-IBS, and 5 to 10% of patients with IBS have been previously infected with *Giardia* (Horman et al., 2004a,b; Grazioli et al., 2006; Hanevik et al., 2009; Dizdar et al., 2010; Allain and Buret, 2020). In a cohort study conducted following the outbreak of giardiasis in Bergen, Norway, 80.5% of patients developed symptoms consistent with PI-IBS (Hanevik et al., 2009). However, the mechanisms responsible for this phenomenon warrant further investigation. To establish the causality between *Giardia* and PI-IBS, a novel neonatal rat model of intestinal hypersensitivity was developed (Halliez et al., 2016). In this model, 50 days post-infection, after full clearance of the parasite, significantly increased jejunal and rectal visceral hypersensitivity was observed. Upregulation of *c-fos* gene expression, a marker of neuronal activation, was clearly evident in post-clearance infected animals.

One factor underlying visceral hypersensitivity is an increase in intestinal permeability (Zhou et al., 2009). Degradation and rearrangement of tight junctional proteins such as Zo-1, claudin-1, and claudin-4 is a hallmark of *Giardia* infection (Allain and Buret, 2020). Paracellular bacterial translocation is observed in giardiasis during the acute phase of infection and persists during the post-infectious state (Halliez et al., 2016). Previous research also showed that *Giardia* infection in mice causes persistent tight junctional damage and bacterial translocation during the acute phase and after trophozoite clearance (post-infective phase; Chen et al., 2013). Bacterial translocation was associated with increased neutrophil infiltration, increased myeloperoxidase activity, and elevated mucosal levels of pro-inflammatory cytokines such as IFN*γ*, TNFα, and IL-1β, during both the acute and post-infective phases (Chen et al., 2013). In addition, alterations to the mucus barrier as a result of mucin degradation and altered mucus production and secretion during infection may further enhance barrier dysfunction (Amat et al., 2017) Thus, bacteria-driven inflammation may be perpetuated even after parasite clearance, which may contribute to development of post-infectious intestinal disorders (Chen et al., 2013; Singer et al., 2019).

Small intestinal bacterial overgrowth (SIBO) is another hallmark of IBS (Ghoshal and Gwee, 2017). Duodenal bacterial overgrowth in association with bile salt deconjugation has been reported in subjects with symptomatic giardiasis, possibly leading to fat malabsorption (Tandon et al., 1977; Muller and von Allmen, 2005). In an attempt to correlate SIBO to post-infectious giardiasis, patients with gastrointestinal disorders following a *Giardia* outbreak were tested with a lactulose breath test, which provides a measurement of hydrogen and methane levels. This study failed to show differences between patients, indicating that giardiasis may not necessarily be associated with SIBO (Morken et al., 2008).

## Conclusion

Disruption of the host intestinal microbiome is well documented in a variety of enteropathogenic infections, and recent evidence has indicated critical roles for the microbiome in modulating disease outcome. During *G. duodenalis* infections, alterations to the composition and functions of the host commensal microbiota have been demonstrated, including an alteration of bacterial species composition and diversity, as well as a direct disruption of intestinal bacterial biofilm structure, which can be associated with the release of pathobionts. Important discoveries on the role of the intestinal microbiota in *Giardia* pathogenesis have been made through the use of germ-free and antibiotic-treated animal models. In addition, by modulating host immunity, mucosal cell function, and the intestinal microenvironment, the microbiome has been shown to be a determining factor in susceptibility or resistance to *Giardia* infection and in disease severity and duration. The development of gnotobiotic animal models will aim to identify the specific roles of individual commensal species in giardiasis, which may represent future therapeutic targets. In addition, microbiota disruption may contribute to development of post-infectious complications including PI-IBS. Further investigation is required to determine how the disruption of homeostatic interactions between microbes and host cells may contribute to chronic intestinal and extra-intestinal complications during giardiasis.

Finally, dietary interventions and the use of probiotics are emerging as potential treatment options for giardiasis as well as other GI diseases in which the gut microbiota is actively implicated. Probiotic microbes may have direct or indirect anti-parasitic effects in the gut and have been shown to reduce the severity and duration of *Giardia* infection. Diet may represent a novel risk factor for development of severe giardiasis and for development of post-infectious complications, particularly in malnourished children. Diet has been shown to influence gut microbiota composition, which may explain in part population specific symptom variability. Future clinical research will be required to determine the efficacy of dietary and probiotic interventions and to identify populations in which these interventions will be most effective. Because *Giardia* has been shown to have both protective and detrimental effects on overall health in human populations and animal models, it will be important to further our understanding of how the microbiome contributes to variations in clinical manifestations.

## Author Contributions

EF, TA, AS, OS, and AB: contribution to manuscript design and writing. All authors contributed to the article and approved the submitted version.

### Conflict of Interest

The authors declare that the research was conducted in the absence of any commercial or financial relationships that could be construed as a potential conflict of interest.

The handling editor declared a past co-authorship with one of the authors AB.
